# Characteristics of immunotherapy trials for nasopharyngeal carcinoma over a 15-year period

**DOI:** 10.3389/fimmu.2023.1195659

**Published:** 2023-08-09

**Authors:** Huageng Huang, Yuyi Yao, Xinyi Deng, Huawei Weng, Zegeng Chen, Le Yu, Zhao Wang, Xiaojie Fang, Huangming Hong, He Huang, Tongyu Lin

**Affiliations:** ^1^Department of Oncology, Sun Yat-sen University Cancer Center, State Key Laboratory of Oncology in South China, Collaborative Innovation Center for Cancer Medicine, Guangdong Key Laboratory of Nasopharyngeal Carcinoma Diagnosis and Therapy, Guangzhou, China; ^2^Department of Dermatology, The First Affiliated Hospital of Guangzhou Medical University, Guangzhou, China; ^3^Department of Oncology, Senior Ward and Phase I Clinical Trial Ward, Sichuan Cancer Hospital and Institute, Sichuan Cancer Center, School of Medicine, University of Electronic Science and Technology of China, Chengdu, China

**Keywords:** nasopharyngeal carcinoma, immunotherapy, Clinicaltrials.gov, clinical trial, immune checkpoint inhibitor

## Abstract

**Background:**

Immunotherapy has been a hotspot in nasopharyngeal carcinoma (NPC) in recent years. This study aimed to provide a comprehensive landscape of the characteristics of immunotherapy clinical trials in NPC and to determine whether contemporary studies are of sufficient quality to demonstrate therapeutic value.

**Methods:**

This is a cross-sectional analysis of NPC trials registered on ClinicalTrials.gov in the last 15 years (Jan 1, 2008-Nov 20, 2022). Only interventional trials with a primary purpose of treatment were included in the final analysis. Characteristics of immunotherapy trials were compared with those of other NPC trials. Chronological shifts in NPC immunotherapy trials were also analyzed.

**Results:**

Of the 440 NPC studies selected, 161 (36.6%) were immunotherapy trials and 279 (63.4%) were other NPC trials. NPC immunotherapy trials were more likely than other NPC trials to be phase 1-2 (82.6% vs. 66.7%, *P* < 0.001), single-arm (51.3% vs. 39.6%, *P* = 0.020), non-randomized (64.8% vs. 44.4%, *P* < 0.001), and enroll fewer than 50 participants (46.3% vs. 34.4%, *P* = 0.015). Blinding was used in 8.8% of NPC immunotherapy trials. Also, 90.7% of NPC immunotherapy trials were recruited nationally and 82.6% were Asia-centric. Although academic institutions and governments (72.7%) were the major sponsors of NPC trials, immunotherapy trials were more likely to be industry-funded than other NPC trials (34.2% vs. 11.5%, *P* < 0.001). The number of NPC immunotherapy trials increased exponentially after 2017, attributed to the exploration of immune checkpoint inhibitors. Immunotherapy combined with chemotherapy was the most commonly investigated regimen.

**Conclusion:**

NPC immunotherapy trials over a 15-year period were predominantly exploratory. To generate high-quality evidence and advance the clinical application of immunotherapy in NPC, more attention and concerted efforts are needed.

## Introduction

Nasopharyngeal carcinoma (NPC) is an Epstein-Barr virus (EBV)-related cancer that is particularly prevalent in South East Asia and Southern China (1). Unlike other head and neck cancers, NPC is susceptible to radiotherapy (RT), which has become the mainstay of treatment for this disease. Despite advances in RT techniques and optimization of chemotherapy regimens, about 20% of patients with locally advanced NPC (LANPC) will recur (2). Moreover, recurrent and/or metastatic (R/M) NPC remains the most serious challenge because the median overall survival (OS) of these patients is only 15.7 months (3). Current conventional treatments, including RT, chemotherapy and surgery, are often accompanied by serious adverse effects and limited efficacy (4). Therefore, there is an urgent need for novel treatment strategies to improve the prognosis of patients with NPC.

In recent years, immunotherapy has sparked a revolution in the clinical management of cancer (5, 6). NPC is regarded as a typical “immune-hot” tumor due to the expression of EBV antigen and CD4^+^/CD8^+^ T-cell target proteins (7, 8), massive lymphocytic infiltration (9), the expression of programmed death ligand-1 (PD-L1) up to 89-95% (10), and the presence of several key immune molecules (CD40, CD70, CD80, and CD86) that regulate T-cell activation (11). Several clinical studies on NPC immunotherapy have shown early successes (12–14). Nevertheless, aside from individual reports, the overall characteristics of NPC immunotherapy clinical trials and whether contemporary studies are of sufficient quality to demonstrate the therapeutic value of immunotherapy in personalized NPC practice are unclear.

ClinicalTrials.gov, a publicly available registry and results database for human clinical studies, provides the most comprehensive clinical study information worldwide. In 2004, the International Committee of Medical Journal Editors (ICMJE) announced a policy as a prerequisite for publication that requires the registration of clinical trials before enrolling participants (15, 16). As of Nov 20, 2022, ClinicalTrials.gov contains detailed information on more than 430 000 clinical trials conducted in over 200 countries. ClinicalTrials.gov is recognized as a promising information source for facilitating the systematic evaluation of clinical trials (16).

In this study, we examined all of the interventional NPC studies registered on ClinicalTrials.gov in 15 years (Jan 1, 2008-Nov 20, 2022). We compared the fundamental characteristics of NPC trials focusing on immunotherapy with the characteristics of other non-immunotherapy NPC trials, and we evaluated the changes over time.

## Materials and methods

### Data source and selection criteria

**Figure 1:**
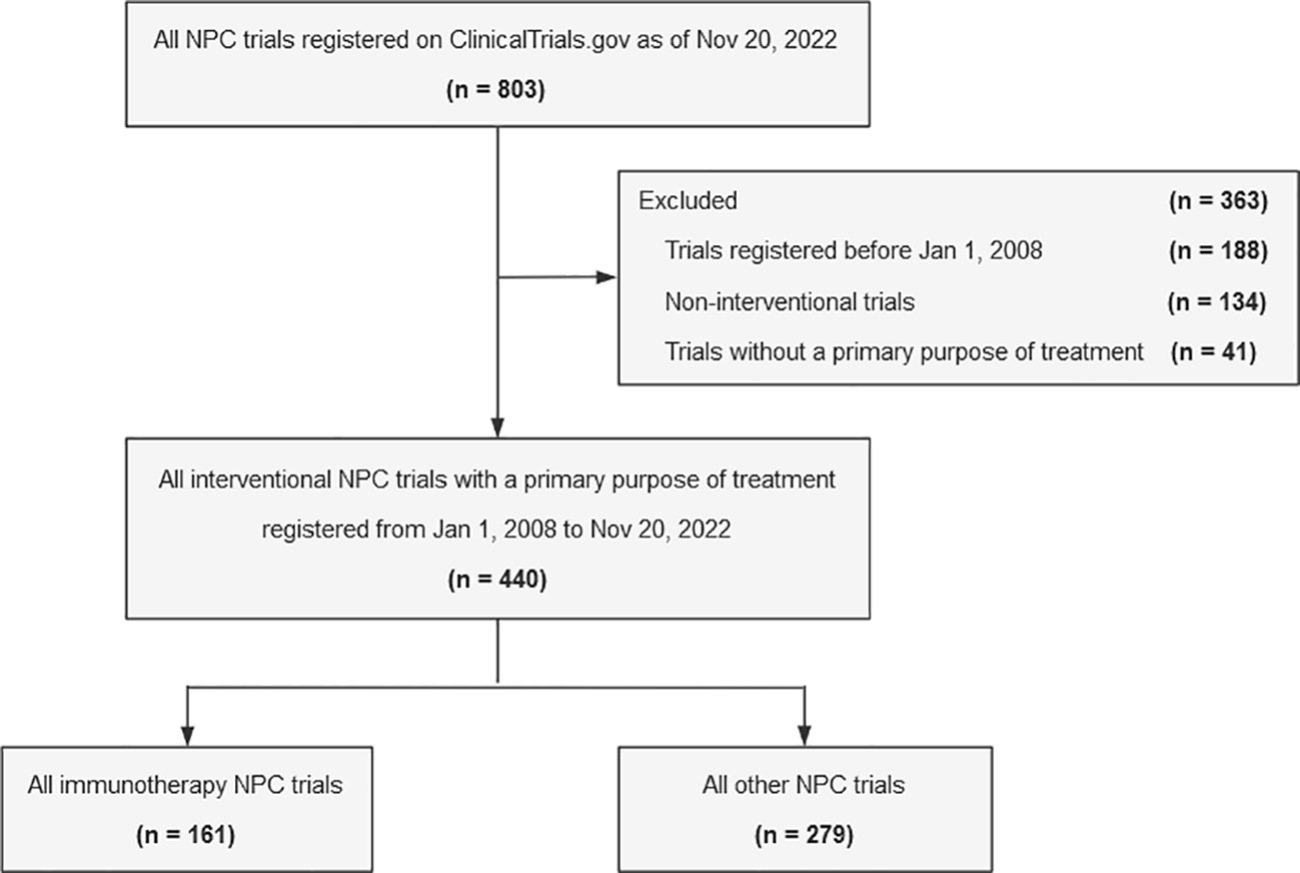
Flowchart identifying trials registered on ClinicalTrials.gov from Jan 1, 2008 to Nov 20, 2022. NPC, nasopharyngeal carcinoma.

This is a cross-sectional analysis of immunotherapy trials for NPC. We searched ClinicalTrials.gov on Nov 20, 2022 using the keyword “*nasopharyngeal carcinoma*”. In total, 803 registered clinical studies were identified and downloaded. We restricted our selection to interventional trials with a primary purpose of treatment that were registered between Jan 1, 2008 and Nov 20, 2022 (n = 440) **(** **Figure 1****)**. This study was considered exempt by the institutional review board of Sun Yat-sen University Cancer Center because it did not involve human participants.

Study data accuracy was ensured by independent verification of all data by three investigators. Two oncologists (H.-G.H. and Y.-Y.Y.) manually and independently reviewed all of the selected trials, and a third author (T.-Y.L.) adjudicated any disagreements. Trials were then categorized according to treatment type, identified by the term “*Intervention/treatment*”, “*Brief Summary*”, or “*Official Title*”. If the treatment type was not clear, other registration information (e.g., “detailed description” and “eligibility”) was reviewed.

We defined immunotherapy trials as studies that (1) added immunotherapy to the standard of care (2); compared any treatment regimens with or without immunotherapy; (3) investigated novel immunotherapy regimens, such as new agents, usages, or dosages; (4) compared different immunotherapy regimens; and (5) evaluated interventions for immunotherapy-related complications. Although EBV-specific monoclonal antibody is a type of immunotherapy tool, some of them work in a more targeted way and partly overlap with ICIs and targeted therapy. Therefore, we excluded EBV-specific monoclonal antibody trials from the immunotherapy study.

Immunotherapy is categorized into four types in this study: (1) immune checkpoint inhibitors (ICIs), including but not limited to programmed cell death protein-1 (PD-1)/PD-L1/cytotoxic T-lymphocyte associated antigen-4 (CTLA-4)/lymphocyte activation gene-3 (LAG-3) inhibitors; (2) adoptive cell therapy (ACT), including adoptive cell transfer of autologous cytotoxic T lymphocytes (CTLs), tumor-infiltrating lymphocytes (TILs), cytokine-induced killer (CIK) cells, and genetically modified cellular immunotherapy such as chimeric antigen receptor-modified T (CAR-T) cell therapy and T cell receptor-engineered T (TCR-T) cell therapy; (3) vaccines; and (4) immunomodulators, including cytokines and oncolytic viruses. The remaining eligible studies constituted the other NPC trials, investigating RT, chemotherapy, surgery, targeted therapy, etc.

### Study variables

We extracted the following information for each trial: (1) whether the trial was registered before participant enrollment; (2) study phase; (3) sample size; (4) number of arms; (5) masking; (6) allocation methods; (7) number of centers; (8) national or international recruitment; (9) age selection; (10) funding source; (11) site location; and (12) recruitment status. As previously described (17–20), if a trial reported only one treatment arm, the allocation methods (if missing) were classified as non-randomized, and the blinding category (if missing) was classified as open-label.

Funding sources were assigned as an industry, National Institutes of Health (NIH), and other academic institutions or governments based on the recorded lead sponsor and/or collaborator for each clinical trial. A trial was classified as industry-funded if its lead sponsor or one of its collaborators was from the industry with no NIH involvement or NIH-funded if its lead sponsor or one of its collaborators was from the NIH with no industry involvement (21). All other trials were classified as other-funded studies.

### Statistical analysis

Descriptive statistics were primarily used to summarize the clinical trial characteristics. Categorical variables were reported as frequencies and percentages. Missing values were excluded from the analyses unless they could be inferred from other relevant data. Trial characteristics were compared using the Pearson χ^2^ test, as well as Fisher’s exact test, if indicated. The statistical significance level was set at *P* < 0.05 (two-sided). Analyses were undertaken using SPSS, version 25.0 (IBM Corp).

## Results

### Characteristics of included trials

Of the 440 NPC trials eligible for analysis, 161 (36.6%) were immunotherapy trials and 279 (63.4%) were other NPC trials **(** **Figure 1****)**.

**Table 1** shows the trial characteristics of immunotherapy and other NPC trials included in this study. Immunotherapy trials were more likely than other NPC trials to be registered before participant enrollment (116 of 161 [72.0%] vs. 128 of 279 [45.9%], *P* < 0.001). In addition, immunotherapy trials tended to have more phase 1-2 studies (133 of 161 [82.6%] vs. 164 of 246 [66.7%], *P* < 0.001) and less likely to be phase 3 studies (23 of 161 [14.3%] vs. 68 of 246 [27.6%], *P* = 0.002) than the other NPC trials. Furthermore, immunotherapy trials were more likely to be single-arm (80 of 156 [51.3%] vs. 109 of 275 [39.6%], *P* = 0.020), non-randomized (103 of 159 [64.8%] vs. 123 of 277 [44.4%], *P* < 0.001), and enroll fewer than 50 participants (74 of 160 [46.3%] vs. 96 of 279 [34.4%], *P* = 0.015) compared with other NPC trials. Blinding was used in 8.8% (14 of 159) of NPC immunotherapy trials and 90.7% (146 of 161) of NPC immunotherapy trials recruited nationally.

**Table 1:** Characteristics of immunotherapy vs. other nasopharyngeal carcinoma trials.

Characteristic	No./Total No. (%)		*P* value^b^
	Immunotherapy NPC trials (n = 161)^a^	Other NPC trials (n = 279)^a^	
Registration before participant enrollment	116/161 (72.0)	128/279 (45.9)	< 0.001
Phase			
Early Phase 1	1/161 (0.6)	2/246 (0.8)	0.002
Phase 1	28/161 (17.4)	26/246 (10.6)	
Phase 1/Phase 2	19/161 (11.8)	14/246 (5.7)	
Phase 2	86/161 (53.4)	124/246 (50.4)	
Phase 2/Phase 3	4/161 (2.5)	7/246 (2.9)	
Phase 3	23/161 (14.3)	68/246 (27.6)	
Phase 4	0/161 (0)	5/246 (2.0)	
Enrollment, No. of patients			
< 50	74/160 (46.3)	96/279 (34.4)	0.040
50 - 100	25/160 (15.6)	61/279 (21.9)	
> 100	61/160 (38.1)	122/279 (43.7)	
No. of study arms			
1	80/156 (51.3)	109/275 (39.6)	0.001
2	59/156 (37.8)	151/275 (54.9)	
≥ 3	17/156 (10.9)	15/275 (5.5)	
Masking			
Open-label	145/159 (91.2)	244/276 (88.4)	0.420
Blind	14/159 (8.8)	32/276 (11.6)	
Allocation			
Randomized	56/159 (35.2)	154/277 (55.6)	< 0.001
Non-randomized	103/159 (64.8)	123/277 (44.4)	
No. of centers			
Single	93/161 (57.8)	183/279 (65.6)	0.102
Multiple	68/161 (42.2)	96/279 (34.4)	
Recruitment			
National	146/161 (90.7)	266/279 (95.3)	0.061
International	15/161 (9.3)	13/279 (4.7)	
Excludes children (aged < 18 y)	150/161 (93.2)	262/279 (93.9)	0.840
Excludes elderly (aged > 65 y)	29/161 (18.0)	65/279 (23.3)	0.227
Funding source			
Industry	55/161 (34.2)	32/279 (11.5)	< 0.001
NIH	9/161 (5.6)	24/279 (8.6)	
Other^c^	97/161 (60.2)	223/279 (79.9)	
Locations^d^			
US/Canada	32/161 (19.9)	40/279 (14.3)	0.142
Europe	10/161 (6.2)	9/279 (3.2)	0.150
Asia	133/161 (82.6)	236/279 (84.6)	0.593
Other^e^	4/161 (2.5)	4/279 (1.4)	0.472
Recruitment status			
Ongoing^f^	118/161 (73.3)	89/279 (31.9)	< 0.001
Stopped early^g^	6/161 (3.7)	24/279 (8.6)	0.075
Completed	19/161 (11.8)	77/279 (27.6)	< 0.001
Unknown	18/161 (11.2)	89/279 (31.9)	< 0.001

NPC, nasopharyngeal carcinoma; NIH, National Institutes of Health; US, United States.

a Different denominators were the number of trials with available data for different variables.

b Calculated using the χ^2^ test or the Fisher exact test if indicated.

c Other Funding sources included individuals, universities, and organizations.

d The sum of the percentages may exceed 100% because categories are not mutually exclusive.

e Other regions included South America, North America other than US/Canada, Central America, Oceania, and Africa.

f This status includes trials that were “not yet recruiting”, “recruiting”, “enrolling by invitation”, “active, not recruiting”, or “suspended” in the database.

g This status includes trials that were “terminated” or “withdrawn” in the database.

**Figure 2:**
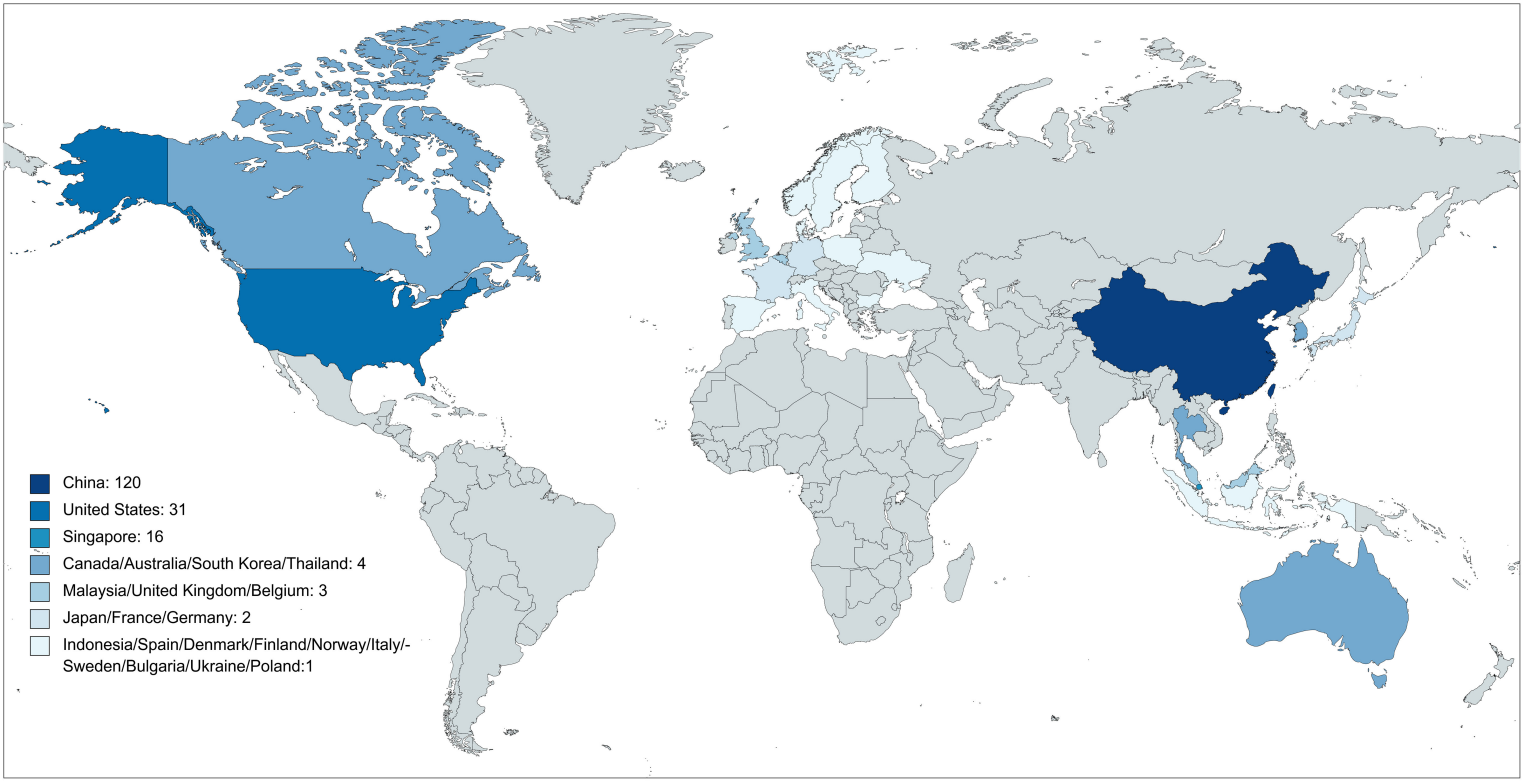
Population distribution of nasopharyngeal carcinoma immunotherapy trials.

Although other funding sources accounted for the highest proportion of immunotherapy and other NPC trials (97 of 161 [60.2%] vs. 223 of 279 [79.9%], *P* < 0.001), immunotherapy trials were more likely to be industry-funded than the other NPC trials (55 of 161 [34.2%] vs. 32 of 279 [11.5%], *P* < 0.001). Asia was the most common study location for the NPC immunotherapy trials (133 of 161 [82.6%]), followed by the United States (US)/Canada (32 of 161 [19.9%]). The most commonly recruited population for NPC immunotherapy trials was distributed in China (120 of 161 [74.5%]), followed by the US (31 of 161 [19.3%]) and Singapore (16 of 161 [9.9%]) (**Figure 2**)

With regard to recruitment status, immunotherapy trials were more likely to be ongoing (118 of 161 [73.3%] vs. 89 of 279 [31.9%], *P* < 0.001) and less likely to be completed (19 of 161 [11.8%] vs. 77 of 279 [27.6%], *P* < 0.001) than other NPC trials. Despite the marginal difference, immunotherapy trials had a lower proportion of trials that stopped early than the other NPC trials (6 of 161 [3.7%] vs. 24 of 279 [8.6%], *P* = 0.075).

### Chronological shifts in the number of NPC immunotherapy trials

**Figure 3:**
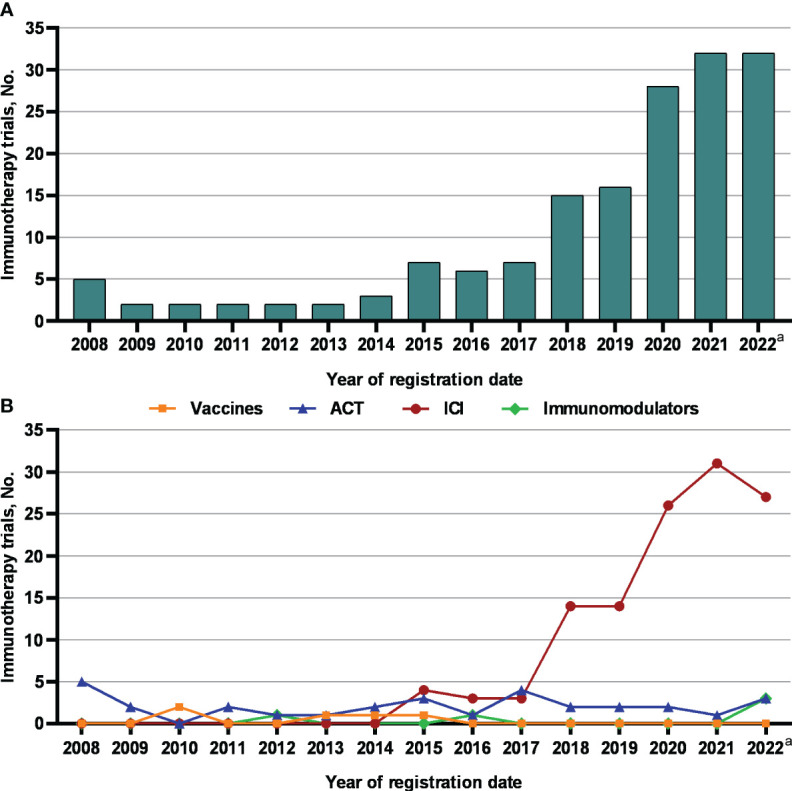
The number of **(A)** immunotherapy trials and **(B)** different types of immunotherapy trials for nasopharyngeal carcinoma registered on ClinicalTrials.gov between 2008 and 2022. ACT, adoptive cell therapy; ICIs, immune checkpoint inhibitors. ^a^ Observed period was Jan 1, 2022 to Nov 20, 2022.

**Figure 3A** shows chronological shifts in the number of NPC immunotherapy trials. Between 2008 and 2017, the number of NPC immunotherapy trials remained relatively stable, ranging between 2 and 7 annually. The number of NPC immunotherapy trials increased from 15 in 2018 to 32 in 2021 (*P* = 0.001). As of Nov 20, 2022, the number of NPC immunotherapy trials in 2022 had reached 32, the same as in 2021.

**Figure 4:**
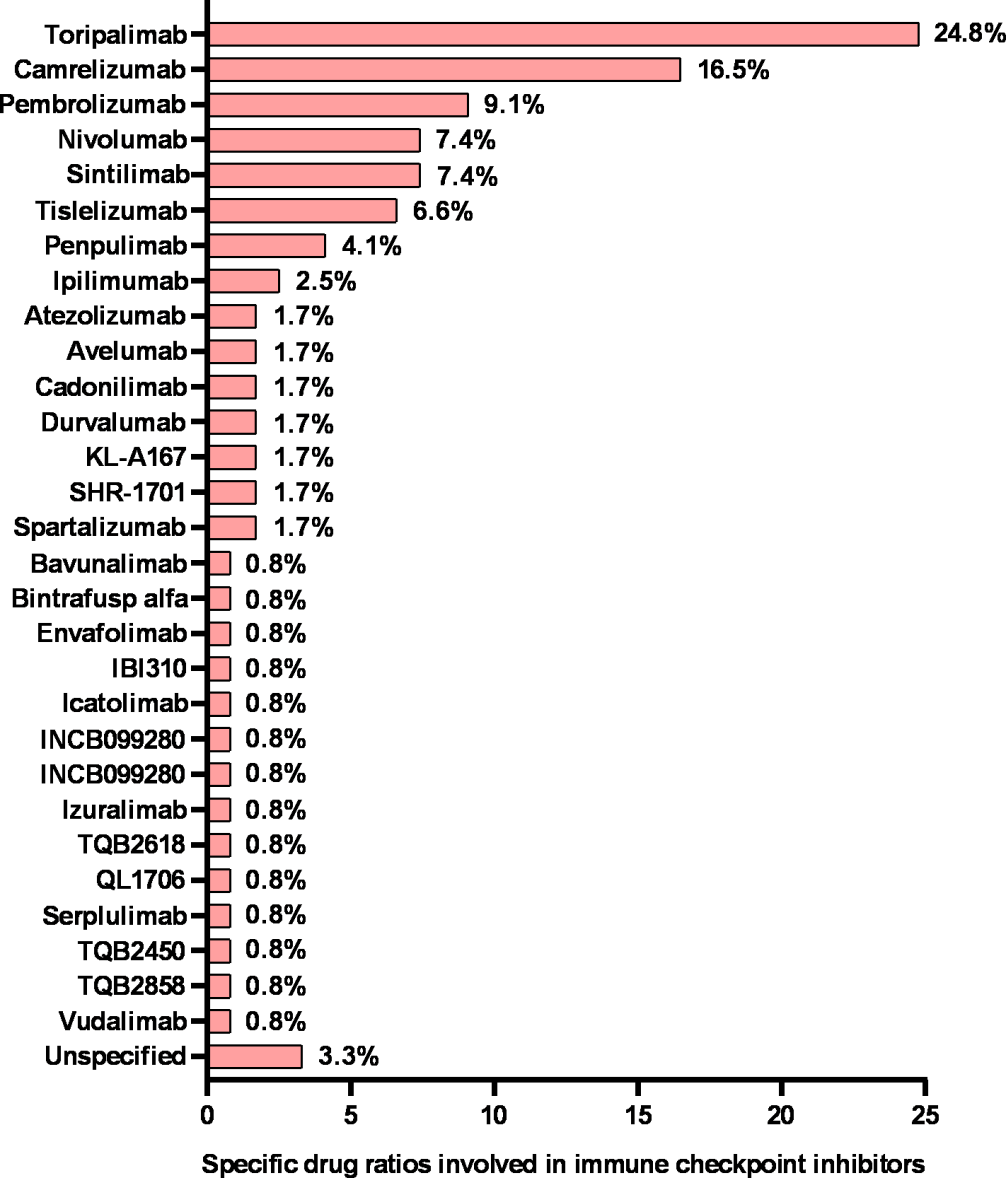
Specific drug ratios in nasopharyngeal cancer clinical trials involving immune checkpoint inhibitors. The sum of the percentages may exceed 100% because categories are not mutually exclusive.

Furthermore, we looked at the chronological shifts in the number of different types of immunotherapy trials for NPC **(** **Figure 3B****)**. From 2008 to 2017, the numbers of ICIs, ACT, vaccine and immunomodulator trials were relatively stable (fewer than 5 annually). Notably, the number of ICI trials rapidly increased to 14 in 2018 and 2019 and doubled to approximately 30 from 2020 to 2022. Toripalimab was the most commonly investigated ICIs in NPC (30 of 121 [24.8%]), followed by camrelizumab (20 of 121 [16.5%]) (**Figure 4**). But the numbers of ACT, vaccine and immunomodulator trials stayed stagnant.

### Chronological shifts in the characteristics of NPC immunotherapy trials

Because NPC immunotherapy trials increased exponentially in number after 2017, we analyzed chronological shifts in the characteristics of NPC immunotherapy trials in the two periods Jan 1, 2008 to Dec 31, 2017 (n = 38, 23.6%) and Jan 1, 2018 to Nov 20, 2022 (n = 123, 76.4%) **(** **Table 2****)**. Compared to 2008-2017, a higher proportion of immunotherapy trials were registered before first participant enrollment in 2018-2022 (94 of 123 [76.4%] vs. 22 of 38 [57.8%], *P* = 0.038). Immunotherapy trials were more likely to be phase 2-3 in 2018-2022 than in 2008-2017 (96 of 123 [78.0%] vs. 17 of 38 [44.7%], *P* < 0.001). Despite the marginal difference, more immunotherapy trials had a sample size of more than 100 patients in 2018-2022 than in 2008-2017 (52 of 123 [42.3%] vs. 9 of 37 [24.3%], *P* = 0.055). The two periods’ basic trial characteristics remained unchanged (all *P* > 0.05).

**Table 2:** Trend changes in characteristics of immunotherapy trials for nasopharyngeal carcinoma registered on ClinicalTrials.gov between Jan 1, 2008 to Dec 31, 2017, and Jan 1, 2018 to Nov 20, 2022.

Characteristic	No./Total No. (%)		P value^b^
	2008-2017 (n = 38)^a^	2018-2022 (n = 123)^a^	
Registration before participant enrollment	22/38 (57.8)	94/123 (76.4)	0.038
Phase			
Early Phase 1	0/38 (0)	1/123 (0.8)	0.003
Phase 1	13/38 (34.2)	15/123 (12.2)	
Phase 1/Phase 2	8/38 (21.1)	11/123 (9.0)	
Phase 2	15/38 (39.5)	71/123 (57.7)	
Phase 2/Phase 3	0/38 (0)	4/123 (3.2)	
Phase 3	2/38 (5.2)	21/123 (17.1)	
Enrollment, No. of patients			
< 50	25/37 (67.6)	49/123 (39.8)	0.012
50-100	3/37 (8.1)	22/123 (17.9)	
> 100	9/37 (24.3)	52/123 (42.3)	
No. of study arms			
1	18/33 (54.6)	62/123 (50.4)	0.813
2	11/33 (33.3)	48/123 (39.0)	
≥ 3	4/33 (12.1)	13/123 (10.6)	
Masking			
Open-label	35/36 (97.2)	110/123 (89.4)	0.194
Blind	1/36 (2.8)	13/123 (10.6)	
Allocation			
Randomized	9/36 (25.0)	47/123 (38.2)	0.168
Non-randomized	27/36 (75.0)	76/123 (61.8)	
No. of centers			
Single	23/38 (60.5)	70/123 (56.9)	0.693
Multiple	15/38 (39.5)	53/123 (43.1)	
Recruitment			
National	33/38 (86.8)	113/123 (91.9)	0.349
International	5/38 (13.2)	10/123 (8.1)	
Excludes children (aged < 18 y)	32/38 (84.2)	118/123 (95.9)	0.022
Excludes elderly (aged > 65 y)	3/38 (7.9)	26/123 (21.1)	0.089
Funding source			
Industry	8/38 (21.1)	47/123 (38.2)	0.001
NIH	7/38 (18.4)	2/123 (1.6)	
Other^c^	23/38 (60.5)	74/123 (60.2)	
Locations^d^			
US/Canada	16/38 (42.1)	16/123 (13.0)	< 0.001
Europe	5/38 (13.2)	5/123 (4.1)	0.057
Asia	22/38 (57.9)	111/123 (90.2)	< 0.001
Other^e^	0/38 (0)	4/123 (3.3)	0.574
Recruitment status			
Ongoing^f^	8/38 (21.1)	110/123 (89.4)	< 0.001
Stopped early^g^	2/38 (5.2)	4/123 (3.3)	0.627
Completed	16/38 (42.1)	3/123 (2.4)	< 0.001
Unknown	12/38 (31.6)	6/123 (4.9)	< 0.001

NIH, National Institutes of Health; US, United States.

a Different denominators were the number of trials with available data for different variables.

b Calculated using the χ^2^ test or the Fisher exact test if indicated.

c Other Funding sources included individuals, universities, and organizations.

d The sum of the percentages may exceed 100% because categories are not mutually exclusive.

e Other regions included South America, North America other than US/Canada, Central America, Oceania, and Africa.

f This status includes trials that were “not yet recruiting”, “recruiting”, “enrolling by invitation”, “active, not recruiting”, or “suspended” in the database.

g This status includes trials that were “terminated” or “withdrawn” in the database.

The number of industry-funded NPC immunotherapy trials increased marginally from 8 of 38 studies (21.1%) in 2008-2017 to 47 of 123 studies (38.2%) in 2018-2022 (*P* = 0.077), but the number of NIH-funded immunotherapy trials decreased significantly from 7 of 38 studies (18.4%) in 2008-2017 to 2 of 123 studies (1.6%) in 2018-2022 (*P* = 0.001). The proportion of other-funded immunotherapy trials remained stable at approximately 60%. In terms of study locations, there was a significant decrease in US/Canada centric from 16 of 38 studies (42.1%) in 2008-2017 to 16 of 123 studies (13.0%) in 2018-2022 (*P* < 0.001) but a significant increase in Asia centric from 22 of 38 studies (57.9%) in 2008-2017 to 111 of 123 studies (90.2%) in 2018-2022 (*P* < 0.001).

### Immunotherapy usage in NPC trials

Among the 161 NPC immunotherapy trials, 46 (28.6%) evaluated single agents and 115 (71.4%) were designed to investigate immunotherapy combination strategies **(** **Table 3****)**. Monotherapy (34 of 46 [73.9%]) was the most commonly explored immunotherapy regimen in single usage, followed by immunotherapy maintenance after standard treatment (11 of 46 [23.9%]). The highest proportion of immunotherapy combination strategies investigated was combination chemotherapy (39 of 115 [33.9%]), followed by radiochemotherapy (27 of 115 [23.5%]) and targeted therapy (19 of 115 [16.5%]).

**Table 3:** Immunotherapy usage in nasopharyngeal carcinoma clinical trials.

Characteristics	No./Total No. (%)
Single usage	46/161 (28.6)
Monotherapy	34/46 (73.9)
Versus ST	1/46 (2.2)
Maintenance after ST	11/46 (23.9)
Combined usage^a^	115/161 (71.4)
IT + CT	39/115 (33.9)
IT + RT	7/115 (6.1)
IT + surgery	2/115 (1.7)
IT + TT	19/115 (16.5)
Multiple IT combination	9/115 (7.9)
IT + CT + RT	27/115 (23.5)
IT + CT + surgery	2/115 (1.7)
IT + CT +TT	8/115 (7.0)
IT + CT + RT + TT	2/115 (1.7)

ST, standard treatment; IT, immunotherapy; CT, chemotherapy; RT, radiotherapy; TT, targeted therapy.

a Trials combining immunotherapy with other therapies simultaneously.

## Discussion

Well-designed clinical trials are desperately needed to validate the clinical applications of immunotherapy in NPC, given its promising efficacy. However, with an overall low incidence rate worldwide for its unique epidemiology, NPC does not attract much attention from most research. To the best of our knowledge, this is the first study assessing the critical characteristics of NPC immunotherapy trials over a 15-year period. By evaluating a comprehensive landscape, we found that NPC immunotherapy trials were predominantly phase 1-2 trials of limited sample size and tended to be single-arm, non-randomized and industry-funded. Blinding was rarely used. Asia was the major study location and clinical trials with international collaboration were lacking as well. The number of NPC immunotherapy trials increased exponentially after 2017, attributed to the exploration of ICIs. But the progress in trial design over time was slow and the basic trial characteristics largely remained unchanged. These findings raise concerns that trials evaluating the therapeutic role of immunotherapy in NPC may not be received the attention or efforts necessary to generate high-quality data. As a result, this orientation toward a less robust design may compromise evidence-based care for NPC.

As an EBV-associated malignancy, NPC is frequently infiltrated with varied stromal cells, making its microenvironment a highly heterogeneous and suppressive harbor that protects NPC cells from drug penetration and immune attack and promotes tumor progression (22, 23). This general immune landscape of NPC renders patients suitable for immunotherapy. In the past 15 years, immunotherapy trials accounted for 36.6% of all NPC trials. Unfortunately, 82.6% of NPC immunotherapy trials were phase 1-2 studies and tended to be single-arm, non-randomized, and enrolled less than 50 participants. Actually, the high proportion of single-arm, non-randomized, early-phase studies may either be because these studies are exploratory, hypotheses generating to fuel future randomized trials involving more patients, or because they were studying more highly innovative expensive cellular-based studies where funding was often inadequate for larger studies with more patients. In addition, the well-defined geographic distribution of NPC might further limit clinicians from conducting large-scale immunotherapy trials. Similarly, Xu et al. (24) tracked the evolving landscape of global immuno-oncology trials in 2007-2019 and found that most immunotherapy trials worldwide were phase 2 studies. Fortunately, NPC immunotherapy trials in 2018-2022 were more likely to be phase 2-3 (78.0% vs. 44.7%, *P* < 0.001) and had a sample size of more than 100 patients (42.3% vs. 24.3%, *P* = 0.055) than in 2008-2017. However, the other basic trial characteristics did not improve in an obvious manner over time.

Establishing international collaborative groups to foster research networks is an effective way to enroll more participants and improve the power of a study. However, 90.7% of NPC immunotherapy trials were conducted in only one region without sufficient international collaboration. Furthermore, in contrast to the findings that the US leads global immunotherapy research with stable growth (24), Asia (82.6%) is the major study location for NPC immunotherapy trials. And the proportion of US/Canada-centric decreased from 42.1% to 13.0% (*P* < 0.001) while the proportion of Asia-centric increased from 57.9% to 90.2% (*P* < 0.001) over the two periods. It’s not surprising because the Asian centricity is concordant with the unique epidemiology of NPC as a predominantly Asian disease. The patterns of NPC (incidence, histology) are different in South East Asia and the rest of the world. It would be helpful if clinical trials could address this discrepancy in future study designs.

Generally, the lengthy duration and high cost of immunotherapy trials may suppress industry enthusiasm. However, our findings showed that NPC immunotherapy trials were more likely to be industry-funded compared with other NPC trials (34.2% vs. 11.5%, *P* < 0.001) and the proportion has increased over time (21.1% vs. 38.2%, *P* = 0.077). It indicates a large market potential in the field of NPC immunotherapy, thus raising financial interests and industrial enthusiasm for sponsorship of such trials. Moreover, 60.2% of NPC immunotherapy trials were other-funded, and this proportion has remained stable over time. It implies that academic institutions and governments continue to play an important role in supporting immunotherapy clinical research for NPC and shoulder vital public health responsibility. Still, allocating more resources to NPC immunotherapy from all relevant parties is essential to improve the effective leveraging of the constrained resources.

It is noteworthy that there was an increasing number of NPC immunotherapy trials after 2017. Actually, this reflects more recent successes in other major tumor types, and therefore there is an increasing interest in studying this intervention in an EBV-related tumor type like NPC that does not have many mutational targets. However, only the number of ICI trials increased significantly, while the numbers of ACT, vaccine and immunomodulator trials remained stagnant. A potential explanation is that ICIs as pan-cancerous antitumor agents were found to have equally promising efficacy in NPC, thus spurring enthusiasm for research. Furthermore, the recognition of ICI-based immunotherapy by the 2018 Nobel Prize in Physiology or Medicine might have further increased researchers’ interest in the exploration of ICIs in NPC. In contrast, the exploration and further application of EBV-specific ACTs and vaccines and immunomodulators were hampered by the lack of specific and effective targets, generally low and transient immune responses, technical limitations and financial shortages. According to the results of published NPC studies (**Supplementary Table 1**), ICIs monotherapy achieves 17.1-34.0% of the objective response rate in the second or later-line treatment of R/M NPC (14, 25–30). In the first-line treatment, the addition of ICIs to chemotherapy also significantly improved progression-free survival and OS in R/M NPC (31–33). Further studies are needed to assess the therapeutic value of ICIs in LANPC and early-stage disease. Notably, CAR-T/TCR-T cell therapy and antibody-drug conjugates may be another promising immunotherapy modality for NPC, as they have shown promising efficacy in a variety of other cancers (34, 35). Therefore, concerted efforts by oncologists, sponsors and other concerned parties are still needed to advance the development of immunotherapy for NPC.

Integration with conventional treatment modalities is one of the trends in immunotherapy. In this study, we found that the most commonly investigated immunotherapy regimen in NPC was combination chemotherapy, followed by combination radiochemotherapy. A recently published study reported on the promising antitumor activity and a manageable toxicity profile of immunotherapy combined with antiangiogenic therapy in R/M NPC (36). In addition, future NPC studies could consider more novel combination strategies to enhance the clinical responses, for example, ICIs combined with ACT (37) or CAR-T cell therapy combined with the oncolytic virus (38).

Limitations of this study should also be acknowledged. First, not all investigators choose ClinicalTrials.gov to register their projects. There are many alternative registries available around the world (39). Nevertheless, ClinicalTrials.gov is the most robust database to date, accounting for 70–80% of the unique clinical trials recorded by the World Health Organization (39). Second, partial NPC trials have not yet been registered on ClinicalTrials.gov, which hindered us from more fully reflecting current global trends in NPC immunotherapy trials. Third, the National Library of Medicine, which operates ClinicalTrials.gov, is unable to validate all registered data. The accuracy of the data relies on the study sponsor. Fourth, we did not include noninterventional trials in our analysis.

In conclusion, NPC Immunotherapy trials over a 15-year period have been largely exploratory. Advancing the clinical application of immunotherapy in NPC requires more attention and concerted efforts to improve the quality of trials.

## Data availability statement

Publicly available datasets were analyzed in this study. This data can be found here: https://clinicaltrials.gov/.

## Author contributions

TL had full access to all of the data in the study and takes responsibility for the integrity of the data and the accuracy of the data analysis. HGH, YY, and XD contributed equally to this work. Concept and design: HGH, YY, XD, HH, and TL. Acquisition, analysis, or interpretation of data: HGH, YY, XD, and TL. Drafting of the manuscript: HGH, YY, and XD. Critical revision of the manuscript for important intellectual content: All authors. Statistical analysis: HGH and XD. Obtained funding: HH and TL. Administrative, technical, or material support: HH and TL. Supervision: HH and TL. All authors contributed to the article and approved the submitted version.
