# Supplementary material for: Characteristics of immunotherapy trials for nasopharyngeal carcinoma over a 15-year period
Source: Front Immunol. 2023 Aug 9;14:1195659. doi: 10.3389/fimmu.2023.1195659 (PMC10445486; doi:10.3389/fimmu.2023.1195659)
Supplement: Supplementary file 1 [file Table_1.docx]

Supplementary Table 1. Published clinical trials of immune checkpoint inhibitors in recurrence and/or metastatic nasopharyngeal carcinoma.

| **Setting** | **Identifier** | **Phase** | **Treatment** | **Sample size** | **ORR (%)** | **Median PFS (months)** | **Median OS (months)** | **Grade ⩾ 3 TRAEs (%)** |
| --- | --- | --- | --- | --- | --- | --- | --- | --- |
| First-line | NCT03121716 | I | Camrelizumab + GP | 24 | 91.0 | NR | NA | 87.0 |
|  | NCT03707509 (CAPTAIN-1st) | III | Camrelizumab + GP | 263 | 87.3 | 9.7 | Immature | 94.0 |
|  | NCT03581786 (JUPITER-02) | III | Toripalimab + GP | 289 | 77.4 | 11.7 | Immature | 89.0 |
|  | NCT03924986 (RATIONALE-309) | III | Tislelizumab + GP | 263 | 69.5 | 9.6 | NR | 18.3 |
| Second-line | NCT02721589 | I | Camrelizumab | 93 | 34.0 | 5.6 | NA | 16.0 |
|  | NCT02915432 (POLARIS-02) | II | Toripalimab | 190 | 20.5 | 1.9 | 17.4 | 14.2 |
|  | NCT02605967 | II | Spartalizumab | 122 | 17.1 | 1.9 | 25.2 | 16.8 |
|  | NCT04586088 | II | Camrelizumab + apatinib | 58 | 65.5 | 10.4 | NR | 58.6 |
| Third- or later-line | NCT02054806 (KEYNOTE-028) | Ib | Pembrolizumab | 27 | 25.9 | 6.5 | 16.5 | 29.6 |
|  | NCT02339558 (NCI-9742) | II | Nivolumab | 44 | 20.5 | 2.8 | 17.1 | 22.0 |
|  | NCT03558191 (CAPTAIN) | I | Camrelizumab | 156 | 28.2 | 3.7 | 17.4 | 15.4 |
|  | NCT03848286 | II | KL-A167 | 132 | 26.5 | 2.8 | 16.2 | 15.0 |

Abbreviations: ORR, objective response rate; PFS, progression-free survival; OS, overall survival; TRAE, treatment-related adverse effect; NA, not available; GP, gemcitabine plus cisplatin; NR, not reached.
